# Supplementary material for: Opposing Roles of apolipoprotein E in aging and neurodegeneration
Source: Life Sci Alliance. 2019 Feb 13;2(1):e201900325. doi: 10.26508/lsa.201900325 (PMC6374993; doi:10.26508/lsa.201900325)
Supplement: Supplementary file 1 [file LSA-2019-00325_Supplemental_Information_1.docx]

**STATISTICAL ANALYSES**

For all the main figures of the manuscript, the exact p values are indicated, as well as the statistical analyses performed.

For all significant analyses, the Confidence Interval is also indicated.

**Analysis 1: Percentages of “responders” and “off-responders” between genotypes (Fig. 2)**

**Outcome measure: Percentage of "responding" cells**

Kruskal-Wallis test P-value: 0.0682 (NS).

**Outcome measure: Percentage of "off-responding" cells**

Kruskal-Wallis test P-value: 0.0062.

| **genotype** | **Lower 95% CI of mean** | **Upper 95% CI of mean** |
| --- | --- | --- |
| APP | 10.09 | 28.47 |
| APOEnull | 1.342 | 7.685 |
| APP-APOEnull | 2.042 | 8.719 |
| Wild-type | 0.6776 | 11.11 |

Post-hoc Dunn's multiple comparisons test

| **Dunn's multiple comparisons test** | **Significance (p<0.05)** |
| --- | --- |
| WT vs. APPPS | * |
| WT vs. APOEnull | ns |
| WT vs. APPPS/APOEnull | ns |
| APPPS/ vs. APOEnull | * |
| APPPS/ vs. APPPS/APOEnull | * |
| APOEnull vs. APPPS/APOEnull | ns |

**Analysis 2: DSI and OSI between genotypes and age groups (Fig. 3 and Supplementary Fig. 4)**

**Outcome measures: log_adjusted dsi and log_adjusted osi**

| **outcome** | **Effect** | **ProbF** |
| --- | --- | --- |
| log_adjusted_dsi | Genotype | 0.0013 |
| log_adjusted_dsi | Age | 0.0325 |
| log_adjusted_osi | Genotype | 0.0076 |
| log_adjusted_osi | Group | 0.0068 |

**Test 1: Linear mixed model was fitted to the DSI and OSI values after log transformation: effect of the genotype and age group**

**Test 2: Comparison of OSI and DSI between wild-type and the other genotype groups**

| **outcome** | **Genotype** | **Effect** | **P value** | **Lower 95% CI of mean** | **Upper 95% CI of mean** |
| --- | --- | --- | --- | --- | --- |
| log_adjusted_dsi | APOEnull | Genotype | 0.1001 | -0.3674 | 0.03219 |
| log_adjusted_dsi | APP | Genotype | 0.0002 | -0.5949 | -0.1599 |
| log_adjusted_dsi | APP-APOEnull | Genotype | 0.4091 | -0.2606 | 0.1062 |
| log_adjusted_dsi | Wild-t | Genotype | . | . | . |

| **outcome** | **Genotype** | **Effect** | **P value** | **Lower 95% CI of mean** | **Upper 95% CI of mean** |
| --- | --- | --- | --- | --- | --- |
| log_adjusted_osi | APOEnull | Genotype | 0.0294 | -0.4613 | -0.02434 |
| log_adjusted_osi | APP | Genotype | 0.0008 | -0.5339 | -0.1393 |
| log_adjusted_osi | APP-APOEnull | Genotype | 0.1584 | -0.3473 | 0.05664 |
| log_adjusted_osi | Wild-t | Genotype | 0 | . | . |

**Test 3: Pair-wise comparison of DSI and OSI in adult and aged mice as compared with age-matched wild-type mice**

| **outcome** | **Age Group** | **Genotype** | **P-value** | **Lower 95% CI of mean** | **Upper 95% CI of mean** |
| --- | --- | --- | --- | --- | --- |
| log_adjusted_dsi | adult | APP | 0.011 | -0.6074 | -0.07877 |
| log_adjusted_dsi | adult | APOEnull | 0.14 | -0.502 | -0.01177 |
| log_adjusted_dsi | adult | APP-APOEnull | 0.2438 | -0.2601 | 0.06615 |
|  |  |  |  |  |  |
| **outcome** | **Age Group** | **Genotype** | **P-value** | **Lower 95% CI of mean** | **Upper 95% CI of mean** |
| log_adjusted_dsi | aged | APP | 0.022 | -0.6298 | -0.0491 |
| log_adjusted_dsi | aged | APOEnull | 0.7494 | -0.4226 | 0.3043 |
| log_adjusted_dsi | aged | APP-APOEnull | 0.9617 | -0.4388 | 0.4178 |

**DSI**

**OSI**

| **outcome: OSI (adults)** | **Age Group** | **Genotype** | **P-value** | **Lower 95% CI of mean** | **Upper 95% CI of mean** |
| --- | --- | --- | --- | --- | --- |
| log_adjusted_osi | adu | APP | 0.0012 | -0.5257 | -0.1298 |
| log_adjusted_osi | adu | APOEKO | 0.2086 | -0.4005 | 0.08754 |
| log_adjusted_osi | adu | APP-APOEnull | 0.5303 | -0.2879 | 0.1483 |
|  |  |  |  |  |  |
| **outcome: OSI (aged)** | **Age Group** | **Genotype** | **P-value** | **Lower 95% CI of mean** | **Upper 95% CI of mean** |
| log_adjusted_osi | old | APP | 0.0422 | -0.7405 | -0.01334 |
| log_adjusted_osi | old | APOEnull | 0.04 | -0.824 | 0.04356 |
| log_adjusted_osi | old | APP-APOEnull | 0.084 | -0.6735 | 0.07638 |

**Test 4: Pair-wise comparison of DSI and OSI between adult and aged mice for each genotype**

**DSI**

| **outcome: DSI** | **genotype** | **P-value** | **Lower 95% CI of mean** | **Upper 95% CI of mean** |
| --- | --- | --- | --- | --- |
| log_adjusted_dsi | APP | 0.0445 | -0.03514 | 0.4431 |
| log_adjusted_dsi | APOEnull | 0.9654 | -0.2714 | 0.2597 |
| log_adjusted_dsi | APP-APOEnull | 0.141 | -0.04932 | 0.3464 |
| log_adjusted_dsi | Wild-type | 0.1513 | -0.07173 | 0.4631 |
| **OSI** |  |  |  |  |
| **outcome: OSI** | **genotype** | **P-value** | **Lower 95% CI of mean** | **Upper 95% CI of mean** |
| log_adjusted_osi | APP | 0.1303 | -0.04926 | 0.3823 |
| log_adjusted_osi | APOEnull | 0.1094 | -0.06338 | 0.6278 |
| log_adjusted_osi | APP-APOEnull | 0.0371 | 0.01623 | 0.5247 |
| log_adjusted_osi | Wild-type | 0.3951 | -0.1276 | 0.3229 |

**Analysis 3: Amyloid burden and plaque density between APP and APP/APOEnull mice (Fig. 4)**

**Outcome measure: Amyloid load (assessed by IHC)**

Unpaired t-test P-value: 0.8709 (N.S.).

**Outcome measure: Amyloid load (assessed by Methoxy)**

Unpaired t-test P-value: <0.0001.

| **genotype** | **Lower 95% CI of mean** | **Upper 95% CI of mean** |
| --- | --- | --- |
| APP | 7.223 | 11.23 |
| APP-APOEnull | 2.07 | 5.111 |

**Outcome measure: Amyloid density (assessed by IHC)**

Unpaired t-test P-value: 0.1764 (N.S.).

**Outcome measure: Amyloid density (assessed by Methoxy)**

Unpaired t-test P-value: 0.0004.

| **genotype** | **Lower 95% CI of mean** | **Upper 95% CI of mean** |
| --- | --- | --- |
| APP | 331 | 480.1 |
| APP-APOEnull | 100.7 | 296.7 |

**Analysis 4: Differences between the levels of TBS soluble, FA soluble and oligomeric Aß (Fig. 4)**

**Outcome measure: TBS soluble Aß40**

Mann-Whitney test P value: 0.0169

| **genotype** | **Lower 95% CI of mean** | **Upper 95% CI of mean** |
| --- | --- | --- |
| APP | 97.81 | 255.2 |
| APP-APOEnull | 61.79 | 101.5 |

**Outcome measure: TBS soluble Aß42**

Unpaired t-test P-value: <0.0001

| **genotype** | **Lower 95% CI of mean** | **Upper 95% CI of mean** |
| --- | --- | --- |
| APP | 69.21 | 25.13 |
| APP-APOEnull | 61.79 | 46.62 |

**Outcome measure: FA Aß40**

Mann-Whitney test P value: 0.0004

| **genotype** | **Lower 95% CI of mean** | **Upper 95% CI of mean** |
| --- | --- | --- |
| APP | 1606 | 5758 |
| APP-APOEnull | -13.49 | 1180 |

**Outcome measure: FA Aß42**

Unpaired t-test P-value: 0.019

| **genotype** | **Lower 95% CI of mean** | **Upper 95% CI of mean** |
| --- | --- | --- |
| APP | 1441 | 2773 |
| APP-APOEnull | 734 | 1668 |

**Outcome measure: oligomeric Aß**

Unpaired t-test P-value: 0.0005

| **genotype** | **Lower 95% CI of mean** | **Upper 95% CI of mean** |
| --- | --- | --- |
| APP | 0.06465 | 0.02623 |
| APP-APOEnull | 0.1223 | 0.04996 |

**Analysis 5: Pre-and post-synaptic density between genotypes (Fig. 5)**

**Outcome measure: PSD95 puncta density**

Two-way ANOVA (variables: proximity to plaques and genotype) followed by a Tukey’s post-hoc test.

| **genotype** | **Lower 95% CI of mean** | **Upper 95% CI of mean** |
| --- | --- | --- |
| Wild-type | 8.836e+008 | 1.070e+009 |
| APOEnull | 9.204e+008 | 1.045e+009 |
| APP (close) | 6.061e+008 | 7.593e+008 |
| APP (far) | 9.202e+008 | 1.077e+009 |
| APP-APOEnull (close) | 6.839e+008 | 1.019e+009 |
| APP-APOEnull (close) | 9.134e+008 | 9.555e+008 |

**Outcome measure: Synapsin puncta density**

Two-way ANOVA (variables: proximity to plaques and genotype) followed by a Tukey’s post-hoc test.

| **genotype** | **Lower 95% CI of mean** | **Upper 95% CI of mean** |
| --- | --- | --- |
| Wild-type | 9.675e+008 | 1.197e+009 |
| APOEnull | 8.984e+008 | 1.080e+009 |
| APP (close) | 6.372e+008 | 8.116e+008 |
| APP (far) | 9.506e+008 | 1.110e+009 |
| APP-APOEnull (close) | 7.728e+008 | 1.134e+009 |
| APP-APOEnull (close) | 8.989e+008 | 1.041e+009 |

**Analysis 6: Stereology analyses of the density of dystrophies, microglial cells and astrocytes associated with amyloid (Fig. 5 and Fig. 6)**

**Outcome measure: Density of dystrophies**

Mann-Whitney test P value: 0.043

| **genotype** | **Lower 95% CI of mean** | **Upper 95% CI of mean** |
| --- | --- | --- |
| APP | 3485 | 11279 |
| APP-APOEnull | 1421 | 2995 |

**Outcome measure: Density of microglia**

Mann-Whitney test P value: 0.0006

| **genotype** | **Lower 95% CI of mean** | **Upper 95% CI of mean** |
| --- | --- | --- |
| APP | 2857 | 4093 |
| APP-APOEnull | 1197 | 2062 |

**Outcome measure: Density of astrocytes**

Mann-Whitney test P value: 0.0012

| **genotype** | **Lower 95% CI of mean** | **Upper 95% CI of mean** |
| --- | --- | --- |
| APP | 2349 | 4370 |
| APP-APOEnull | 909 | 1790 |

**Analysis 7: Excitatory and inhibitory neurotransmitter receptors accordingly to the genotypes (Supp. Fig. 8)**

**Outcome measure: Densitometry ratio NMDAR1/GAPDH**

Kruskal-Wallis test P-value: 0.0873 (N.S).

**Outcome measure: Densitometry ratio NMDAR2A/GAPDH**

Kruskal-Wallis test P-value: 0.0619 (N.S).

**Outcome measure: Densitometry ratio NMDAR2B/GAPDH**

Kruskal-Wallis test P-value: 0.1557 (N.S).

**Outcome measure: Densitometry ratio GABA(A)/GAPDH**

Kruskal-Wallis test P-value: 0.2813 (N.S).

**Outcome measure: Densitometry ratio GABA(B)R1a/GAPDH**

Kruskal-Wallis test P-value: 0.052.

**Outcome measure: Densitometry ratio GABA(B)R1b/GAPDH**

Kruskal-Wallis test P-value: 0.0709
